# Reverse rendezvous endoscopic retrograde cholangiopancreatography after liver partition and portal vein ligation

**DOI:** 10.1055/a-2885-8287

**Published:** 2026-07-13

**Authors:** Khurum Hakeem, Eoin Keating, Khalid Bashir, Umair Kamran, Yiannis Skarparis, Dhya Al-Leswas, John Leeds

**Affiliations:** 1 Department of Gastroenterology 105565 Freeman HospitalNewcastle upon TyneTyne and wearUnited Kingdom of Great Britain and Northern Ireland; 2 Department of GastroenterologyThe Northumbria HospitalNorthumberlandEnglandUnited Kingdom of Great Britain and Northern Ireland; 3 Department of Radiology 105565 Freeman HospitalNewcastle upon TyneTyne and wearUnited Kingdom of Great Britain and Northern Ireland; 4 Department of Surgery 105565 Freeman HospitalNewcastle upon TyneTyne and wearUnited Kingdom of Great Britain and Northern Ireland

A 45-year-old-man was diagnosed with primary rectal cancer during colonoscopy. Despite the initial disease response to neoadjuvant chemotherapy, imaging demonstrated progression of the right hepatic metastatic disease. Associated liver partition and portal ligation surgery (ALPPS) was planned. Stage 1 ALPPS was successful but extended hepatectomy and stage 2 ALPPS was complicated by a liver remnant anchor failure requiring repeat surgical intervention. High outputs from the right liver bed drain and cross-sectional imaging identified a liver bed bile leak secondary to a left hepatic duct stricture.

Conventional endoscopic retrograde cholangiopancreatography (ERCP) was unable to access the intrahepatic system due a blind-ending proximal common bile duct (CBD). 1​ Subsequent percutaneous transhepatic cholangiography (PTC) drainage accessed the left intrahepatic duct but could not traverse the stricture to the CBD.

Multidisciplinary team discussion recommended a “reverse-rendezvous ERCP” to attempt internal drainage ( Video 1 ). 2​ ​3​ Initial CBD cannulation and pressure cholangiograms demonstrated persistent occlusion of the proximal CBD. Anterograde cholangiogram via the PTC demonstrated no communication of the intrahepatic ducts with the CBD, with a transition zone at the left hepatic duct.

**Video 1** Reverse rendezvous ERCP for postoperative bile leak after ALPPS. Endoscopic and percutaneous guidewires were advanced into the liver bed collection, creating through-and-through access from the ampulla to the left intrahepatic ducts. Following balloon dilatation, a plastic stent was placed for internal drainage, with contrast confirming flow into the duodenum.


**Fig. 1:**
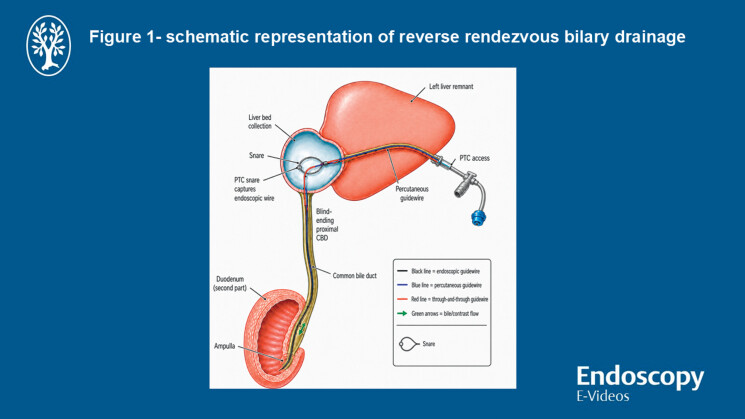
Schematic representation of reverse rendezvous bilary drainage.

Endoscopic wire manipulation and occlusion cholangiography successfully accessed the left liver bed collection. Simultaneous percutaneous guidewire manipulation resulted in both wires entering the collection. A snare was deployed via the PTC and captured the endoscopic guidewire. This created a through-and-through guidewire crossing the ampulla, traversing the left intrahepatic duct and exiting the PTC site ( Fig. 1 ).

The tract was dilated by a 6 mm dilatation balloon. A 10 French×15 cm straight plastic stent was deployed entering the left intrahepatic ductal system. A PTC drain was deployed “kissing” the proximal CBD stent. PTC contrast injection demonstrated flow entering the duodenum. Four weeks following the initial rendezvous procedure, the drain was successfully internalised via the mature tract.

This case represents the first published “reverse-rendezvouz ERCP” in a patient with unique post-operative biliary anatomy.

Endoscopy_UCTN_Code_CPL_1AK_2AD.
